# Supplementary material for: Genome-Wide Identification of Common Bean PvLTP Family Genes and Expression Profiling Analysis in Response to Drought Stress
Source: Genes (Basel). 2022 Dec 16;13(12):2394. doi: 10.3390/genes13122394 (PMC9777604; doi:10.3390/genes13122394)
Supplement: Supplementary file 1 [file genes-13-02394-s001.zip › Table S2.docx]

**Table S2**  Primer sequences of *PvLTP* genes used for qRT-PCR

| Gene | Forward primer (5'-3') | Reverse primer (5'-3') |
| --- | --- | --- |
| *PvLTPI*-*27* | CAGCAGGGTGTTGTAGCGG | AGGGACATTGACATGGCAGA |
| *PvLTPI*-*28* | GGCGATGTGACAACGGAC | TCCAGCAGCACCCAGAAT |
| *PvLTPI*-*41* | GCCTGAGCTACCTAAGAACCG | AACCCTGGGCTAACGCAC |
| *PvLTPI*-*42* | CCGTGCGTAACATTCCTTCA | CCTGAGCATTATTGGGATTGAG |
| *PvLTPI*-*44* | TCGTTGCCGTGCTGTGCT | GGTCTGGGGTGGTCTTTGC |
| *PvLTPI*-*45* | AAGGCACAAGCACAGATGACA | GCAGCACTGAGCAGGAACA |
| *PvLTPII*-*3* | CACTTTGGTGGTGCTGCTTGT | AGGTTTGATGGAGGAGTTGAAGAG |
| *PvLTPV*-*1* | TGGCTTGTTGTAGCAGAGTTGG | TGGAATGGTGATAGCAGTGGC |
| *PvLTPV*-*2* | GGAGGGTGTTGTGAAGTTTGC | TCATCTTGTGCTGCTGAGGAA |
| *Actin* | GAAGTTCTCTTCCAACCATCC | TTTCCTTGCTCATTCTGTCCG |
