# Supplementary figures and images for: Genome-Wide Identification of Common Bean PvLTP Family Genes and Expression Profiling Analysis in Response to Drought Stress
Source: Genes (Basel). 2022 Dec 16;13(12):2394. doi: 10.3390/genes13122394 (PMC9777604; doi:10.3390/genes13122394)

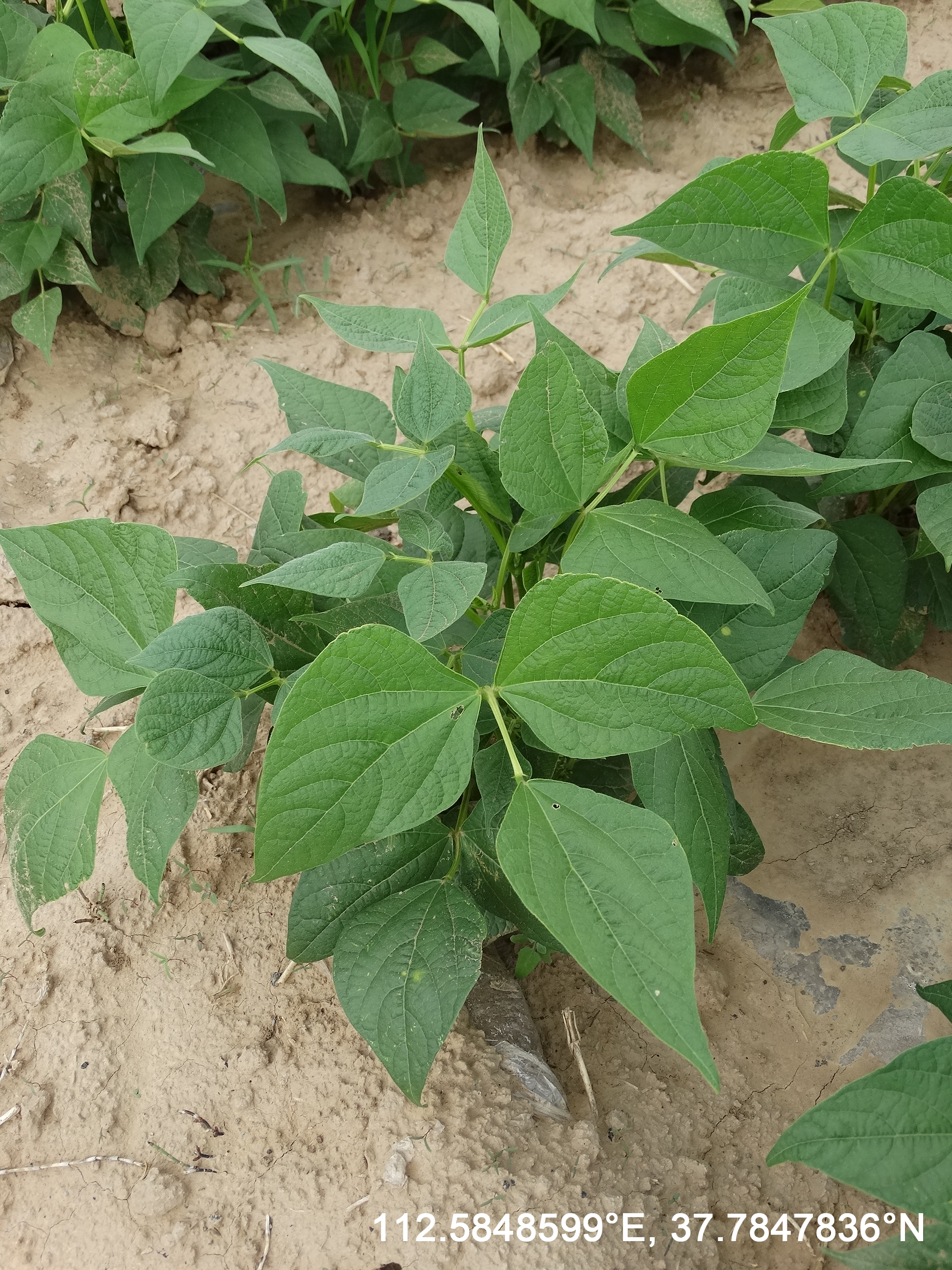

Supplement: Supplementary file 1 [file genes-13-02394-s001.zip › Figure S1.tif]

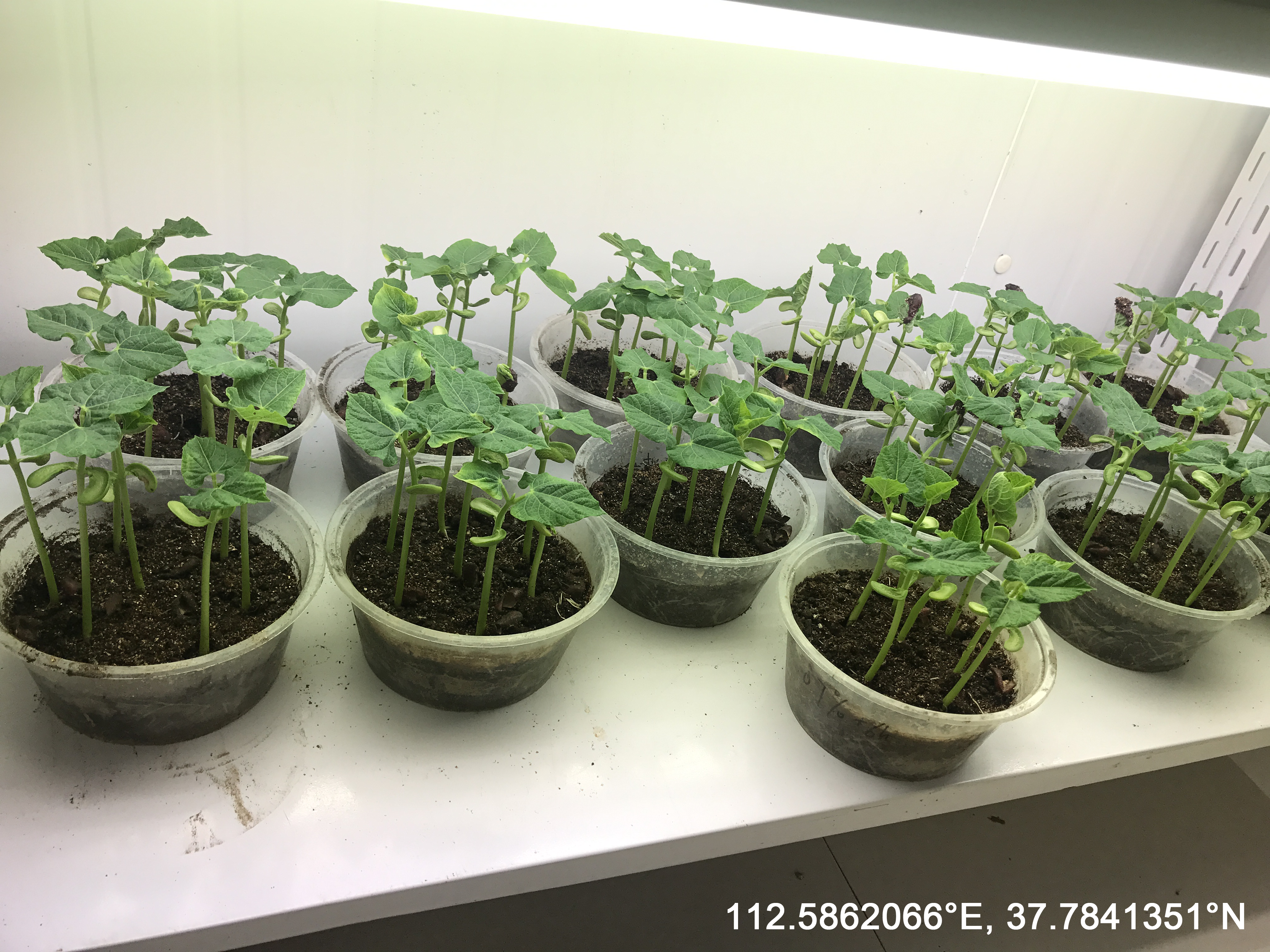

Supplement: Supplementary file 1 [file genes-13-02394-s001.zip › Figure S2.tif]
